# Dietary diversity and other predictors of low birth weight in Gurage Zone, Ethiopia: Prospective study

**DOI:** 10.1371/journal.pone.0300480

**Published:** 2024-04-30

**Authors:** Teshome Gensa Geta, Samson Gebremedhin, Saad Ahmed Abdiwali, Akinyinka O. Omigbodun

**Affiliations:** 1 Department of Public Health, College of Medicine and Health Science, Wolkite University, Wolkite, Ethiopia; 2 School of Public Health, College of Health Science and Medicine, Addis Ababa University, Addis Ababa, Ethiopia; 3 Department of Public Health, College of Health Science and Medicine, Gollis University, Hargeisa, Somaliland; 4 Pan African University (PAU), Life and Earth Science Institute (including Health and Agriculture), University of Ibadan, Ibadan, Oyo State, Nigeria; 5 Department of Obstetrics & Gynaecology, College of Medicine, University of Ibadan, Ibadan, Oyo State, Nigeria; Debre Berhan University, ETHIOPIA

## Abstract

**Background:**

Low birth weight (LBW) is a major public health problem in Ethiopia. Dietary diversity is a key indicator of maternal dietary adequacy that may affect birth weight but little is known about their relationship. Hence, this study aimed to assess the association of suboptimal maternal dietary diversity during pregnancy and low birth weight in Gurage Zone, Ethiopia.

**Methods:**

The prospective study was conducted among 1062 pregnant women enrolled consecutively in between 16 to 20 gestational weeks and followed until delivery. The baseline data were collected at recruitment and dietary diversity was assessed using the minimum dietary diversity score for women (MDD-W) tool in three different rounds. The average of three scores was considered to categorize women into optimal (consumed ≥ 5 food groups) and suboptimal (consumed < 5 food groups) dietary diversity groups. The risk of low birth weight among suboptimal dietary diversity was assessed using modified Poisson regression with robust standard error.

**Results:**

Of the 1062 pregnant women recruited, 959 (90.4%) women completed follow-up. Among them, 302 (31.5%) women are having optimal and the rest, 657 (68.5%) women are having suboptimal dietary diversity. The risk of low birth weight was significantly higher among women with sub-optimal dietary diversity than among those with optimal diversity (ARR = 1.89, 95% CI: 1.25, 2.84). Other factors such as rural residence (ARR = 1.61, 95% CI: 1.43, 1.87), age > = 35 years (AAR = 3.94, 95% CI: 2.41, 6.46), being underweight (ARR = 1.81, 95% CI: 1.14, 2.86), height < 150cm (ARR = 4.65, 95% CI: 2.52, 8.59), unwanted pregnancy (ARR = 3.35, 95% CI: 2.23, 5.02), preterm delivery (3.65, 95% CI: 2.27, 5.84) and lack of nutritional counseling (ARR = 1.69, 95% CI: 1.08, 2.67) significantly increased the risk of low birth weight.

**Conclusion:**

Suboptimal dietary diversity associated low birth weight. Promoting dietary diversity by strengthening nutritional education and avoiding unwanted pregnancy particularly among rural residents may help to reduce the incidence of low birth weight.

## Introduction

Low birth weight (LBW) has been defined by World Health Organization (WHO) as weight at birth less than 2500g [1]. It is an important public health indicator, a summary measure of multifaceted public health problems including unfavorable socio-demographic conditions, long-term maternal malnutrition, ill health and poor pregnancy care [1–3].

Low birth weight contributes to poor health outcomes for a baby. It predisposes newborns to hypoglycemia, hypothermia and other neonatal morbidities. Hence, it is one of the major determinants of perinatal survival and infant mortality [4, 5]. Infants with low birth weight have a higher risk of developing chronic diseases such as hypertension, obesity and diabetes in adult life compared to infants with normal birth weight [6, 7]. It is also closely associated with long-term sequels like neurodevelopmental impairment including mental retardation and learning disabilities [8, 9].

The overall global prevalence of low birth weight is estimated to be 15%, representing more than 20 million births a year [10]. There is considerable variation in prevalence across the regions. It is highly prevalent in low- and middle-income countries, with 95.6% of overall low birth weight occurring in developing countries [11]. The regional estimate of low birth weight includes 28% in South Asia and 13% in sub-Saharan Africa. In these regions, more than half of new-borns were not weighted at birth which may result in an underestimation of low birth weight [10]. The nationwide prevalence of low birth weight in Ethiopia from EDHS is 13.2% with high variation among different regions of the country [12, 13].

The factors responsible for low birth weight (LBW) are yet to be completely understood, even though many studies have been conducted to ascertain the underlying factors [14]. Socio-demographic factors such as residence, family income, occupation, mother’s age and other maternal attributes such as short stature, illness, inadequate maternal nutrition, short birth interval and high parity were found to be associated with low birth weight [11, 15–17]. The predisposing factors to LBW differ from one setting to the other. In developed regions LBW is mainly associated with prematurity, high maternal age and smoking whereas in developing regions, it is primarily associated with restricted fetal growth linked to inadequate maternal nutrition [18, 19].

Maternal nutrition has profound effects on fetal growth, development and subsequent newborn birth weight. The interactions between different micronutrients deficiency in intake of inadequate dietary diversity might be associated with low birth weight [20, 21]. Although it is important to isolate the interaction of specific nutrients or food with birth weight, it might be inadequate to explain the complex pattern of consumption [22] and the interaction between nutrients [23]. Therefore, dietary diversity, the proxy measure of nutrient adequacy and overall diet quality in women [24, 25], might be useful in understanding the relationship between overall maternal nutrition adequacy during pregnancy and low birth weight [26]. Dietary diversity is the number of food groups consumed over 24 hours [24]. Studies indicate dietary diversity during pregnancy is significantly associated with birth outcomes [27–31]. The overall improvement in dietary diversity significantly decreases the risk of having a small for gestational age baby [32–34].

Studies conducted in the western and north-eastern parts of Ethiopia have shown that inadequate dietary diversity during pregnancy was significantly associated with preterm delivery and LBW [35, 36]. In contrast, studies in Ghana and Pakistan suggested that inadequate dietary diversity during pregnancy had no significant association with LBW [21, 37]. In view of the contrasting findings and the paucity of studies on the relationship between dietary diversity and low birth weight, this study was conducted to determine the association between maternal dietary diversity during pregnancy and low birth weight in Gurage Zone, Ethiopia.

## Methods

### Study setting

The study was conducted in the Gurage zone; one of the zones in the southern nation’s nationalities and people’s regions (SNNPR) of Ethiopia. The administrative center of the Zone is Wolkite town, located 158 km southwest of Addis Ababa; the capital of Ethiopia. It is a densely populated zone with a total population of 3,567,377 with an area of 5,893.40km^2^ according to the 2015 census [38] and comprising 14 districts and 5 town administrations. The zone has one general hospital, six primary hospitals, 72 health centers, and 412 health posts. According to a 2019 report, the proportion of health center to population ratio is 1: 26,983 which is slightly less than the national standard of 1:25,000 [39].

### Study design, study period and population

The prospective study was conducted from Jan 2021 to Oct 2021 among pregnant women attending the ANC center. All pregnant women attending the first ANC visit during the second trimester (16 to 20 weeks), who planned to complete subsequent routine ANC visits and were planning to give birth at the selected health facilities were included as the participants. Women with multiple pregnancies, known medical conditions, terminally ill during data collection, and who were not permanent residents of the area were excluded from the study.

### Sample size determination

The sample size was calculated using open Epi Fleiss statistical software available at http://www.openepi.com/SampleSize/SSCohort.htm by considering 95% confidence level (2-sided), and 80% power. The ratio of exposed (suboptimal dietary diversity) and unexposed (optimal dietary diversity) was assumed to be 2:1 based on results from a previous study [40]. The proportion of low birth weight among women having sub-optimal dietary diversity was 15.6 [27] and its proportion from the general population was 8.1% [41]. Thus, we considered an 8% difference in proportion among those women having optimal and suboptimal dietary diversity. By using these parameters, the calculated sample size was 714. The final sample size after considering the design effect of 1.5 and adding 20% non-response rate was 1062.

### Sampling procedure

A multistage cluster sampling method was used by dividing the study area into rural districts and semi-urban town administrations. Six rural districts and two town administrations were randomly selected. Then, the sample size was allocated proportionally to randomly selected health facilities in each selected districts and town administration.

The study participants were recruited consecutively until sample size was filled and followed up to delivery. At enrollment, all pregnant women were in their second trimester (16 to 20 gestational weeks) attending first ANC visit. Sub-optimal dietary diversity of women was measured three times during follow-up and the average of three different measurements was considered for final grouping into two cohorts (optimal and sub-optimal dietary diversity). The participants having average dietary diversity (MDD-W) greater than or equal to five food groups were categorized as optimal dietary diversity and those with dietary diversity (MDD-W) less than five food groups were categorized as suboptimal dietary diversity based on FAO guideline on minimum dietary diversity for women (MDD-W) [24].

### Variables of the study

The dependent variable of the study was birth weight; categorized as low birth weight (< 2500 g) and adequate birth weight (≥ 2500 g). The health care provider attending delivery measured the birth weight of the baby with very light clothing by the surface-mounted baby weight measurement scale within 24 hours after delivery. The independent variables were the socio-demographic profile of participants, maternal dietary practice during pregnancy including meal frequency, meal skip, and avoidance of certain food items, and obstetric histories such as parity, birth interval, presence of danger signs, and pregnancy intention (wanted and planned or unwanted and unplanned pregnancy).

The other independent variable was household food insecurity assessed using the household food insecurity access scale (HFIAS) guideline [42]. The HFIAS covers a recall period of the preceding 30 days and consists of nine occurrences (yes/no) questions and nine "frequency-of-occurrence" questions (rarely, sometimes, or often). Based on the responses, households were categorized as food secure and mildly, moderately and severely food insure as per guideline [43].

The minimum dietary diversity of women was the main independent variable measured by using a standard FAO questionnaire containing 10 food groups [24]. Women were asked about their diet in the preceding 24 hours whether or not they consumed from ten food groups (grains, white roots and tubers; pulses; nuts and seeds; dairy; meats, poultry and fish; eggs; dark green leafy vegetables; another vitamin A-rich fruits and vegetables; other vegetables; and, other fruits). Each food group consumed (scored 1) and not consumed (scored 0) were summed up to a score ranging from 0 to 10. The dietary scoring was done in the three different rounds; 16 to 20 weeks, 28 to 29 weeks and 36 to 37 weeks of gestation, during follow up and the average of three scores was used to define overall dietary diversity. Women who consumed food items from less than 5 groups were categorized as sub-optimal dietary diversity and those who consumed food items from 5 and above groups were categorized as optimal dietary diversity [24, 43].

### Anthropometric measurements

Height was measured by carder HM 200P portable stadiometer which is composed of a ruler and sliding headpiece. During the measurement, the patient stands erect with the back, buttocks, and heels against the tape. The nearest higher centimeter measurement was taken by sliding the headpiece down until it touches the patient’s scalp. The weight of the women was measured at enrollment using a platform beam balance weight scale. Mid-upper arm circumference (MUAC) was measured to assess nutritional status. The measurement was done halfway between the olecranon process of the left arm and the acromion using a non-stretchable tape meter. The MUAC measurement below 23cm was considered undernutrition [44].

The body mass index (BMI) was computed by dividing weight at recruitment (kg) by the square of height in meters. The weight measured at enrollment was considered as a pre-pregnancy weight due to a lack of recorded pre-pregnancy weight and poor reliability of orally reported weight. Women are categorized into four groups based on the BMI measurement; underweight (< 18.5 kg/m^2^), normal (18.5–24.9 kg/m^2^), overweight (25–29.9 kg/m^2^) and obese (≥ 30 kg/m^2^) [45].

### Data collection

The interview administered questionnaire and the checklist were prepared partly by reviewing several articles [35, 46–48] and partly by adopting a standard questionnaire [24, 42, 49]. The questionnaire has different sections including socio-demographics, obstetric history, dietary diversity, nutritional practice, household food security and the checklist comprises gestational age, pre-pregnancy BMI, gestational age at delivery, sex of newborn, and birth weight.

The data were collected in four different phases. The baseline data were collected during recruitment (16 to 20 weeks of gestational), and the assessment of women’s dietary diversity and other medical conditions were repeated from 28 to 29 and 36 to 37 weeks of gestations. Finally, pregnancy outcomes including the status of the baby (alive and dead), sex, gestational age at delivery, and birth weight were taken immediately after birth.

### Data quality assurance

The questionnaire was prepared in English language and translated into the local Amharic language. To have better consistency, it was translated back to English and edited by a person with good knowledge of both languages. It was pre-tested and brief training was given to enumerators before actual data collection. Data collection was closely supervised and completeness was checked on each day of data collection. Incomplete data was traced back and edited accordingly. The anthropometric measurement was taken by trained personnel and the calibration of all equipment was done on a daily basis.

### Data processing and analysis

Data were entered and cleaned using Epi—data version 3.02 statistical software and exported to STATA 14 version. Descriptive statistics such as mean, standard deviation, frequency, and percentage were used to describe the study population and continuous variables were checked for normal distribution using a probability plot and Shapiro Wilk test at p-value > 0.05. The occurrence of multicollinearity among independent variables was ensured using the variance inflation factor at a cut-off point of 10 and there was no multicollinearity.

The baseline difference between women in two groups (optimal and suboptimal dietary diversity) was assessed by using the chi-square test for significance. The risk of occurrence of low birth weight among explanatory variables was estimated by using modified Poisson regression with robust standard error. To determine independent predictors of low birth weight, all variables with a p-value less than 0.25 in the bi-variable analysis were considered for the multivariable modified Poisson regression model. The adjusted risk ratio (ARR) with a 95% confidence interval and p-value < 0.05 were used to declare statistical significance.

### Ethical considerations

Ethical approval was obtained from the institutional review board of Wolkite University, Ethiopia (RCSUILC/059/2013) and the joint Ethics Review Committee of the University of Ibadan and the University College Hospital, Ibadan, Nigeria (UI/EC/20/0463). Written informed consent was obtained from each participant after a brief explanation of all research activities, aim, benefit, and risk of participating in the study. Voluntary participation and confidentiality of data were kept in every stage of data handling and no identifier was included.

## Results

### Sociodemographic characteristics of the participants

Out of the 1062 patients enrolled in the study, 959 (90.4%) completed follow-up, of which 302 (31.5%) were women having optimal dietary diversity and 657 (68.5%) were women having suboptimal dietary diversity. The mean (±SD) age of the participants is 25.62 (±4.89). The majority of participants 610 (63.6%) were urban residents and 434 (45.3%) were Muslims. More than half (68.2%) of women were from the Gurage ethnic group. Regarding the monthly income of a family, 275 (28.7% had an average of less than 2000 ETB per month (Table 1).

**Table 1:** Sociodemographic characteristics of women attending antenatal clinics in the Gurage Zone, South Central Ethiopia, 2021.

Variables	Frequency (%)	Women with MDDW ≥ 5	Women with MDDW < 5	P-value
Age group (years)				
18–24	410 (42.8)	122	288	0.51
25–34	491 (51.2)	163	328	
35 and above	58 (6.0)	17	41	
Residence				
Urban	610 (63.6)	236	374	< 0.001
Rural	349 (36.4)	66	283	
Ethnicity				
Gurage	654 (68.2)	228	426	0.008
Amhara	140 (14.6)	23	107	
Oromo	61 (6.4)	18	43	
Others	104 (10.8)	33	81	
Religion				
Muslim	434(45.3)	138	296	0.09
Orthodox	398 (41.5)	113	285	
Protestant	93 (9.7)	36	57	
Others	34 (3.5)	15	19	
Educational attainment				
No formal education	324 (33.8)	93	231	0.08
Primary education	319 (33.3)	92	227	
Secondary education	211 (22.0)	81	130	
Tertiary education	105 (10.9)	36	69	
Women occupation				
Housewife	546	147	339	0.004
Merchant	117	47	70	
Government employed	120	49	71	
Farmer	105	32	73	
Others	131	57	74	
Marital status				
Married	944 (98.9)	294	650	0.07
Not ever married	15 (1.6)	8	7	
Family size				
1–5	732 (76.3)	222	510	0.16
>5	227 (23.7)	80	147	
Average monthly income (ETB)				
1^st^ quartile (150–2000)	275 (28.7)	55	220	< 0.001
2^nd^ quartile (2001–4000)	226 (23.6)	64	162	
3^rd^ quartile (4001–6000)	232 (24.2)	76	156	
4^th^ quartile (6001–15000)	226 (23.6)	107	119	

ETB: Ethiopian Birr (1USD was equivalent to 32 Ethiopian birrs at a time study)

### Obstetric and anthropometric characteristics of women

At enrollment, all pregnant women were between 16 and 20 weeks of gestational age. Among the participants, 340 (35.5%) were nulliparous, 294 (30.7%) were primiparous and 325 (33.9%) were multi-parous. The majority of pregnancies, 862 (89.9%) were wanted and planned, and the rest, 97 (10.1%) were unwanted and unplanned. Of the total, 530 (55.3%) pregnancies occurred more than 24 months after the end of the previous pregnancy, whereas 139 (14.5%) were within 24 months. There was a history of abortion in 107 (16.4%) of the participants (Table 2).

**Table 2:** Obstetric and anthropometric characteristics of women attending antenatal clinic in Gurage Zone, South Central Ethiopia, 2021.

Variables	Frequency (%)	LBW, n (%)	NBW, n (%)	p-value
**Parity**				
Nulli-parity	340 (35.1)	40 (37.7)	300 (35.2)	0.719
Primi-parity	294 (30.7)	35 (33.0)	259 (30.4)	
Multi-parity	325 (33.9)	31 (29.2)	294 (34.4)	
**Previous Abortion** (n = 671)				
Yes	157 (23.4)	17 (23.3)	140 (23.4)	0.981
No	514 (76.6)	56 (76.7)	458 (76.6)	
**Birth interval** (n = 669)				
Less than 24 months	139 (20.8)	13 (18.6)	126 (21.0)	0.631
24 months and above	530 (79.2)	57 (81.4)	473 (79.0)	
**Pregnancy desirability**				
Wanted and planned	862 (89.9)	81 (76.4)	781 (91.6)	< 0.001
Unwanted and unplanned	97 (10.1)	25 (23.6)	72 (8.4)	
**Any danger sign**				
Present	142 (14.8)	17 (16.0)	125 (14.7)	0.705
Absent	817 (85.2)	89 (84.0)	728 (85.3)	
**Gestational age at delivery**				
Less than 37 weeks	30 (3.2)	11 (10.4)	19 (2.2)	< 0.001
37 weeks and above	922 (96.8)	95 (89.6)	827 (97.8)	
**Sex of baby**				
Female	495 (51.6)	53 (10.4)	442 (89.6)	0.449
Male	464 (48.4)	56 (11.9)	408 (88.1)	
**Maternal height**				
< 150 cm	29 (3.0)	8 (7.5)	21(2.5)	0.004
≥ 150 cm	930 (97.0)	98 (92.5)	832 (97.5)	
**MUAC (cm)**				
< 23	158 (16.5)	16 (16.0)	141 (16.5)	0.894
≥ 23	801 (83.5)	89 (84.0)	711 (83.5)	
**Pre-pregnancy BMI (kg/m** ^**2**^ **)**				
Underweight (< 18.5)	79 (8.2)	17 (16.0)	62 (7.3)	0.001
Normal (18.5–24.9)	695 (72.5)	80 (75.5)	615 (72.1)	
Overweight (25–29.9)	156 (16.3)	8 (7.5)	148 (17.4)	
Obese (≥ 30)	29 (3.0)	1 (0.9)	28 (3.3)	

LBW: Low birth weight, NBW: normal birth weight, n: number of subjects, MUAC: mid-upper arm circumference, BMI: body mass index

Regarding the nutritional status of women, 158 (16.5%) were undernourished (MUAC < 23 cm) and 801 (83.4%) are well-nourished (MUAC ≥ 23 cm). Among the women, 695 (72.5%) had normal pre-pregnancy BMI (18.5–24.9kg/m^2^). The majority, 891 (92.9%), had inadequate gestational weight gain and the rest 68 (7.1%) had adequate gestational weight gain (Table 2).

### Incidence of low birth weight

The overall incidence of low birth weight was 11.1% (95% CI = 9.1%, 13.0%). The incidence of low birth weight was higher among babies born to women with suboptimal dietary diversity (12.8%) than babies born to women with optimal dietary diversity (7.3%) (p < 0.012). The mean (±SD) birth weight of the newborns was 3150.58 ± 409.82 with a range of 2000gm to 4550gm.

### Predictors of low birth weight

Table 3 describes the bi-variable and multivariable modified Poisson regression analysis. The variables with a p-value < 0.25 in bi-variable analysis were considered for a multivariable modified Poisson regression model with robust standard error. After adjusting for covariates, women having sub-optimal dietary diversity had higher risk of low birth weight compared to those having optimal dietary diversity (ARR = 1.89, 95% CI: 1.25, 2.84). Other factors including residence, age greater than 35, unwanted pregnancy, lower monthly income, height less than 150 cm, being underweight (pre-pregnancy BMI < 18.5 kg/m^2^) and preterm delivery were significantly associated with low birth weight (Table 3).

**Table 3:** Bi-variable and multivariable modified Poisson regression analysis of the factors affecting low birth weight among neonates delivered in the Gurage Zone, South Central Ethiopia, 2021.

Variables	Category	Crude Estimate		Adjusted Estimate	
		RR (95% CI)	P value	RR (95% CI)	p-value
Dietary diversity	Optimal	1 (ref)		1(ref)	
	Suboptimal	1.75 (1.12, 2.75)	0.014	1.89 (1.25, 2.84)	0.002*
Residence	Urban	1 (ref)		1(ref)	
	Rural	0.75 (0.51, 1.12)	0.163	1.61 (1.43, 1.87)	0.007*
Age in years	18–24	1.29 (0.90, 1.85)	0.161	0.99 (0.96, 1.04)	0.957
	25–34	1 (ref)		1(ref)	
	35 and above	1.07(1.05, 1.09)	< 0.001	3.94 (2.41,6.46)	< 0.001*
Women occupation	Housewife	1 (ref)		1(ref)	
	Merchant	0.84 (0.42,1.66)	0.616	1.04 (0.55, 1.98)	0.889
	Government employed	1.01(0.53, 1.86)	0.997	1.11 (0.59, 2.11)	0.734
	Farmer	2.6 (1.68, 4.00)	< 0.001	1.29 (0.81,2.05)	0.285
	Others	1.69 (0.92, 3.09)	0.088	1.08 (0.67, 1.73)	0.746
Monthly income in quartiles	1^st^ (150–2000 ETB)	0.68 (0.41,1.13)	0.140	1.48 (1.27, 1.85)	0.011*
	2^nd^ (2001–4000 ETB)	0.59 (0.31, 1.09)	0.058	1.56 (1.33, 1.95)	0.031*
	3^rd^ (4001-6000ETB)	1.24 (0.79, 1.95)	0.344	1.01 (0.98, 1.03)	0.494
	4^th^ (6001–15000 ETB)	1 (ref)		(ref)	
Pregnancy desirability	Planned	1 (ref)		1(ref)	
	Unplanned	2.74 (1.84, 4.07)	< 0.001	3.35 (2.23, 5.02)	< 0.001*
Women height in cm	< 150 cm	2.62 (1.41, 4.86)	0.002	4.65 (2.52, 8.59)	< 0.001*
	≥ 150 cm	1 (ref)		1 (ref)	
Pre-pregnancy BMI (in kg/m^2^)	Underweight (< 18.5)	1.87 (1.19, 2.98)	0.009	1.81 (1.14, 2.86)	0.011*
	Normal (18.5–24.9)	1 (ref)		1 (ref)	
	Overweight (25–29.9)	0.44 (0.22, 0.90)	0.025	0.46 (0.22, 0.94)	0.035*
	Obese (≥ 30)	0.29 (0.04, 2.08)	0.223	0.24 (0.03, 1.91)	0.180
Food security	Food secure	1 (ref)		1 (ref)	
	Mild food insecure	2.79 (1.37, 5.69)	0.005	0.54 (0.27, 1.11)	0.094
	Moderate food insecure	1.02 (0.61, 1.69)	0.943	1.13 (0.72, 1.76)	0.590
Nutritional education	Yes	1(ref)		1 (ref)	
	No	1.90 (1.32, 2.73)	< 0.001	1.69 (1.08, 2.67)	0.022*
Fasting	Yes	1.52 (1.05, 2.19)	0.024	1.68 (1.04, 2.75)	0.035*
	No	1 (ref)		1 (ref)	
Meal skip	Yes	1.61 (1.11, 2.33)	0.012	1.39 (0.97, 1.98)	0.051
	No	1 (ref)		1(ref)	
Gestational age at delivery	Less than 37 weeks	3.55 (2.14, 5.91)	< 0.001	3.65 (2.27, 5.84)	< 0.001*
	37 weeks and above	1 (ref)		1(ref)	

*RR*: *risk ratio*, *CI*: *confidence interval*, *ref*: *reference group*, *ETB*: *Ethiopia birr*, *MUAC*: *mid-upper arm circumference*, *BMI*: *body mass index*, * indicates significant variables

## Discussion

The magnitude and risk factors of low birth weight vary across the socio-economic status of countries and also vary across different regions in the same country. The current prospective study aimed to assess the association of suboptimal dietary diversity and low birth weight in a Gurage Zone, Ethiopia.

The incidence of low birth weight observed in this study was consistent with studies conducted in Tigray [50], Butajira [51], Dessie, Amhara region [52], Gambia [53] and Lusaka, Zambia [54]. It is also consistent with the report from the 2016 Ethiopian Demographic Health Survey (EDHS) (13.2%) [13] but relatively lower compared to studies conducted in Dilla [14], Dire Dawa [55], Tanzania [56], Nigeria [57] and Ghana [58]. Conversely, the proportion of low birth weight in the current study was relatively higher than findings from other studies [43, 59, 60]. This variation might be due to the socio-demographic difference between the study participants and the difference in the quality of maternal health care services and trained health personnel.

The present study revealed that low birth weight was significantly higher among women with suboptimal dietary diversity compared to their counter parts. This is in line with previous studies that have shown suboptimal dietary diversity during pregnancy increases the risk of low birth weight [27, 61, 62]. This could be due to the reason that a high dietary diversity diet in pregnancy provides important micro-nutrients essential for the growth and development of the fetus. Lack of those micro-nutrients might result in intrauterine growth retardation that in turn leads to low birth weight [29]. On contrary, other studies conducted in Uganda [43], showed no association between dietary diversity and low birth weight. The difference might be due to differences in the sociodemographic characteristics of study participants.

Factors such as nutritional education and history of fasting were found to be risk factors for low birth weight. Women who did not get nutrition-related education and counseling were more likely to give birth to low birth weight babies than their counterparts. This report is in line with the finding of previous studies [11, 63]. This is evidenced by the improvement in dietary diversity of pregnant women and also nutritional counseling might help to improve healthcare-seeking behavior that in turn influences fetal wellbeing.

Women having a history of fasting in the current pregnancy had a higher risk of low birth weight compared to those with no history of fasting. The possible explanation might be that fasting women get a lower percentage of total energy intake, macronutrients and micronutrients than none fasting women. The current report was contrary to reports from other studies [64–66]. The difference in the result might be attributed to the nutritional status of study participants, differences in dietary habits, and the duration of fasting time.

We also found that socio-demographic factors including age greater than 35 years, rural residence and lower household income quartiles were significantly associated with low birth weight. Women of advanced age (> 35 years) had a higher risk of low birth weight than women aged 25 to 35 years. Previous studies [59, 67–70] have shown similar results. Although the mechanism of the effect of maternal age on low birth weight is unclear, it is possible that the association of advanced age with chronic diseases and their complications in pregnancy may contribute to low birth weight [71]. Other factors such as epigenetic DNA reprogramming with advanced age [72] and a decline in the quality of women’s egg [73] might also contribute to low birth weight.

Women from rural residences had 1.61 times higher risk of low birth weight than those from urban areas. This is in line with other studies conducted in the Sidama zone, Ethiopia [74] and Iran [60, 70]. This could be due to poor maternal literacy, low accessibility of health services, low maternal health service utilization, and involvement in high-intensity activities in rural areas. In this study, women in the lowest quartile of monthly income showed a higher risk of delivering an infant with low birth weight. Other studies reported similar findings [75, 76]. These statistics could be due to the reason that low family income hinders adequate care during pregnancy in terms of nutrition and health care [76].

Anthropometric factors such as height < 150 cm and pre-pregnancy BMI < 18.5 kg/m^2^ are significantly associated with low birth weight. In this study, women whose height was < 150 cm were 4.65 times more likely to deliver a low birth weight baby compared to women with a height > 150 cm. It is consistent with the report from other studies [50, 55]. This result might suggest that the narrow pelvis and limited intra-uterine space associated with short stature [77, 78] and lower blood flow in limited uterine volume [79] restricts fetal growth. On the other hand, a mother with a pre-pregnancy BMI of less than 18.5 kg/m^2^ had a nearly 2 times higher risk to deliver a low birth-weight baby compared to a normal BMI. Previous studies reported that pre-pregnancy underweight women were more likely than normal-weight women to deliver LBW infants [80, 81]. The possible explanation for this might be BMI less than 18.5kg/m^2^ indicate the presence of chronic under nutrition and lower tissue nutrient reserve. Hence; maternal under-nutrition can hinder the growth and development of the fetus [76].

Obstetric history such as pregnancy desirability and pre-term delivery (birth < 37 weeks of gestation) significantly increased the risk of low birth weight. The current study showed unplanned and unwanted pregnancies had a higher risk of low birth weight than planned and wanted pregnancies. This report is confirmatory to previous studies [4, 82]. This might be due to the reason that unwanted pregnancy could result in risky behaviours such as inadequate prenatal care, increased stress, and reduced social support during pregnancy [82]. This study also showed preterm delivery increased the risk for low birth weight (ARR: 3.65 95% CI: 2.27, 5.84). This is in line with studies conducted in the northern part of Ethiopia [83, 84], Nepal [85], Cameron [67] and Zambia [54]. Pre-term delivery being a predictor of low birth weight is clear that the fetus has not grown enough to achieve its target term weight [67].

### Strength and limitation of the study

The strength of our study was involving prospective study with which pregnant women followed until delivery. During follow up, dietary diversity was measured in three different rounds and average of it was considered. This gives better information than one-time dietary diversity scoring on the previous studies done so far. Nevertheless, some limitations of the study need to take into consideration during the interpretation of the results. Assessing dietary diversity using the minimum dietary of women (MDD-W) tool may have recall bias and the amount of food consumed did not take into consideration.

## Conclusion

The incidence of low birth weight in the Gurage zone is significantly higher among women with suboptimal dietary diversity. Rural residence, maternal age greater than 35, lower monthly income quartile, unwanted and unplanned pregnancy, height less than 150cm, absence of nutritional education, fasting during pregnancy and preterm delivery were the other co-factors for low birth weight. It is recommended to improve dietary diversity among pregnant women to reduce the incidence of low birth weight. The governmental and non- governmental organizations working on maternal health should focus on diversifying diet of pregnant women by giving special attention for women from rural residents with low monthly income and also should have strategic plan for preventing unwanted and unplanned pregnancy.

## Supporting information

S1 Data (XLSX)
